# Increased cardiovascular mortality during the COVID-19 pandemic: do not neglect causality

**DOI:** 10.1093/ehjopen/oead119

**Published:** 2023-11-19

**Authors:** Bente Halvorsen, Pål Aukrust, Tuva Børresdatter Dahl, Ida Gregersen

**Affiliations:** Research Institute of Internal Medicine, Rikshospitalet, Oslo University Hospital and University of Oslo, Sogsvannveien 20, 0372 Oslo, Norway; Research Institute of Internal Medicine, Rikshospitalet, Oslo University Hospital and University of Oslo, Sogsvannveien 20, 0372 Oslo, Norway; Research Institute of Internal Medicine, Rikshospitalet, Oslo University Hospital and University of Oslo, Sogsvannveien 20, 0372 Oslo, Norway; Research Institute of Internal Medicine, Rikshospitalet, Oslo University Hospital and University of Oslo, Sogsvannveien 20, 0372 Oslo, Norway

**Keywords:** COVID-19, Cardiovascular disease, Inflammation

We read with great interest the paper by Lunardi *et al*.,^1^ describing negative long-term effects on survival and quality of life after STEMI, due to reduced cardiovascular health care during COVID-19 lockdown. This falls in line with similar reports from many different countries and highlights important mechanisms behind the cardiovascular consequences of the COVID-19 pandemic. There are, however, also many studies suggesting a direct and causal effect of SARS-CoV-2 infection on cardiovascular disease (CVD) and death, and we believe this causality has been overlooked in Norway and European countries. We emphasize the need to explore this relationship further, to prevent excess CVD and reduce the future burden on the healthcare systems and loss of life in the years to come.

Reports from many countries, and across continents, have shown an increase in deaths from CVD since March 2020, when the COVID-19 pandemic hit globally. For the first time since 1995, an increase in cardiovascular mortality was seen in Norway in 2021 and 2022. A study conducted in the USA also revealed excess cardiovascular death throughout the first 2 years of the pandemic and that these waves virtually coincide with the COVID-19 death waves.

**Figure 1:**
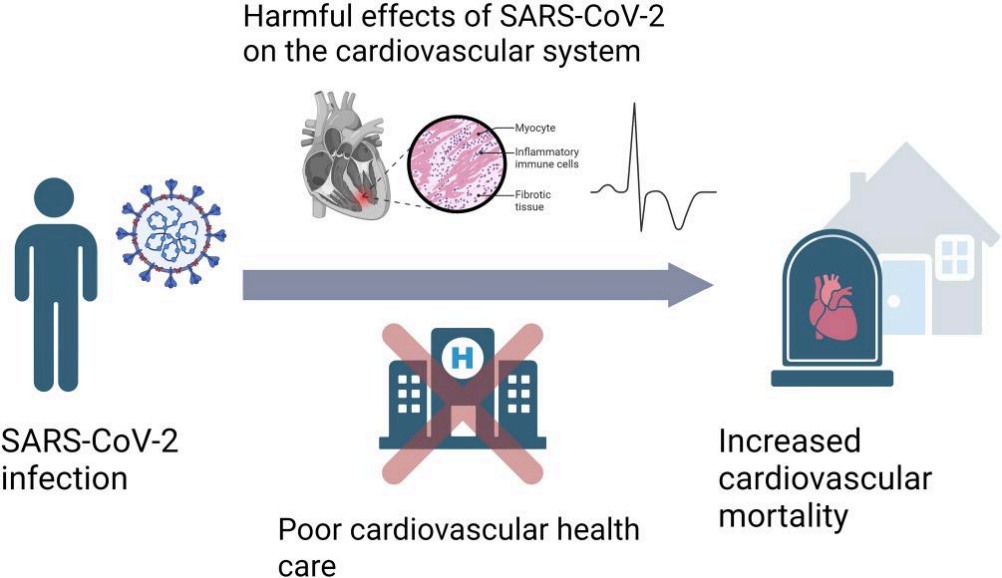
Illustration of potential mechanisms underlying the increased cardiovascular mortality observed during the COVID-19 pandemic. Both direct and indirect effects are likely important. Created with BioRender.

Lunardi *et al*.^1^ illustrate that, due to reduced treatment, patients having a STEMI under lockdown were predicted to lose an average of 1.55–2.03 life-years compared with someone having a STEMI before the pandemic. There is however a complex relationship between CVD and COVID-19, which complicates the interpretation of cardiovascular mortality data. They share many of the same risk factors, and they often coincide, at least in severe COVID-19. Thus, even though it is widely accepted that an important contributor is reduced CVD treatment, as illustrated by Lunardi *et al*.^1^ there are also several lines of evidence pointing to a causal effect of COVID-19 on CVD death. We believe it is a combination of the two (*Figure 1*).

SARS-CoV-2 infection may escalate an already established cardiovascular risk, and early data from China show that the total case mortality rate was almost five times higher in patients with underlying CVD. This increased risk has been confirmed in several studies. The cardiovascular system is broadly affected by SARS-CoV-2 infection, both directly, due to infecting of the heart, causing myocardial inflammation, and indirectly, through increased inflammation, endothelial dysfunction, and increased thrombogenicity, which can all lead to cardiovascular events. A causal interaction is supported by a national US healthcare database study, reporting that COVID-19 increases the risk of 20 different CVDs, including cerebrovascular disorders, ischaemic heart disease, heart failure, and thromboembolic disease, for up to 1 year after infection. The risks were evident regardless of age, race, sex, and other cardiovascular risk factors and importantly included people without any cardiovascular disease prior to COVID-19, supporting a strong association.^2^

We and others have provided data on several potential mechanisms for the causal interaction between COVID-19 and CVD, such as alterations in fibrogenesis, persistently enhanced immune activation, and altered gut microbiome. We recently showed that CXCR6, previously identified as a risk allele for severe COVID-19, is associated with COVID-19 mortality in hospitalized patients, potentially mediated through cardiac involvement. CXCR6 is the receptor for CXCL16, a predictor of CVD both in heart failure and atherosclerosis-driven acute coronary syndromes. The interaction between these mechanisms and cardiovascular death, however, needs further investigation. Another striking finding, and an example of potential causality, is our observation that COVID-19 patients had altered LDL particles 3 months after hospitalization. The LDL particles had imprinted fragments of innate immune activation and antibodies not observed in healthy controls.^3^ These alterations can possibly render the particles more inflammatory and pro-atherogenic, increasing the cardiovascular risk. The fact that the turnover of LDL particles is 2–3 days points to a sustained alteration of the vascular system, including lipoprotein metabolism, in individuals after severe COVID-19. Thus, this combination of persistent inflammation and altered atherogenic LDL particle phenotype, could, particularly in pre-disposed individuals, promote the acceleration of atherosclerosis and potentially other CVDs.

It is still not known how and to what degree COVID-19 increases the risk of cardiovascular death. Even though we acknowledge the importance of reduced cardiovascular health care, as described by Lunardi *et al*., considering the current literature, it is unwise to ignore the possible causality suggested by us and many others. Improved knowledge about the underlying mechanisms is warranted and will provide important tools to prevent CVD and cardiovascular death in COVID-19-affected individuals, as well as will improve our ability to meet new pandemics with amended knowledge.
